# In intergroup conflict, self-sacrifice is stronger among pro-social individuals, and parochial altruism emerges especially among cognitively taxed individuals

**DOI:** 10.3389/fpsyg.2015.00572

**Published:** 2015-05-06

**Authors:** Carsten K. W. De Dreu, D. Berno Dussel, Femke S. Ten Velden

**Affiliations:** ^1^Department of Psychology, University of AmsterdamAmsterdam, Netherlands; ^2^Center for Experimental Economics and Political Decision Making, University of AmsterdamAmsterdam, Netherlands

**Keywords:** intergroup conflict, competition, parochial altruism, dual systems, ego depletion

## Abstract

Parochial altruism is decomposed in a tendency to benefit the in-group along with a tendency to ignore, derogate, and harm rivaling out-groups. Building off recent work suggesting that decisions to cooperate can be relatively fast and intuitive, we examine parochial altruism in intergroup conflict when cognitive deliberation is rendered difficult or not. Predictions were tested in an experiment using an incentivized Intergroup Prisoner’s Dilemma–Maximizing Differences Game with 95 subjects classified as either pro-social or pro-self being randomly allocated to high vs. low impulse-control conditions. Results showed, first of all, that self-sacrificial decisions to contribute were made faster than decisions not to contribute, and that faster decision time associated with more positive expectations of in-group members. Second, we observed that lowering impulse control with a difficult rather than easy Stroop Task increased the amount contributed to a pool that benefited in-group members while harming out-group members; thus reducing deliberation increased parochial altruism. Finally, results replicated earlier work showing that especially pro-social (vs. pro-self) individuals contributed more to the in-group and did not lower their contributions to the between-group pool that benefitted their in-group and, simultaneously, hurt the out-group. This pattern emerged independent of their impulse control. Thus, (in-group bounded) cooperation is more prominent among individuals with strong rather than weak other-regarding preferences. Moreover, the intuitive tendency to cooperate may have evolved in the context of intergroup conflict and therefore is sharp-edged—in-group bounded and including willingness to aggress out-groups.

## Introduction

Humans owe much of their evolutionary success to their strong capacity to create and maintain cohesive groups within which they engage in complex forms of cooperative exchange, negotiate, and trade, innovate, and disseminate knowledge, insights, values, and preferences (Darwin, 1871; Bowles and Gintis, 2004, 2011; Henrich and Henrich, 2007; Wilson, 2012; De Dreu et al., 2014a). In fact, being included in strong, well-functioning, and innovative groups provides fitness functionality to its individual members because they are more likely to survive, prosper, and reproduce than individuals living in groups where most members lack such cooperative inclinations: “…groups with a greater number of courageous, sympathetic, and faithful members, who were always ready to warn each other of danger, to aid and defend each other …would spread and be victorious over other tribes” (Darwin, 1871, p. 156).

Because of the group’s functionality to individual fitness, humans may have evolved a “group psychology” that includes a propensity to (i) identify with groups and its members, (ii) empathize with the needs and interests of fellow group members, (iii) self-sacrifice, trust, and cooperate with other group members, and (iv) loyally commit and contribute to the functioning of one’s group (De Dreu et al., 2014a; De Dreu and Kret, 2015). Furthermore, because groups exist next to other groups with whom they cooperate, compare, and compete, group efficiency often is relative – groups that generate greater surplus than other groups become relatively strong and prosperous, achieve a relatively favorable social status position, and may be better able to exert power and influence over other groups and their members (De Dreu et al., 2014a). Accordingly, the evolved group psychology must be, at least to some extent, relative and comparative vis-à-vis other groups. Throughout evolution humans may have become prepared to self-sacrifice in order to cooperate with others, but especially with those they rely upon, are interdependent with, and expect interactions with in the future, that is, with those others that are perceived to be part of one’s group (Balliet et al., 2014). Some even proposed that such in-group bounded cooperation may have co-evolved with tendencies to aggress against rivaling out-groups (LeVine and Campbell, 1972; Campbell, 1975; Bernhard et al., 2006; Bowles, 2009; De Dreu et al., 2014a). After all, when group efficiency is relative, promoting in-group efficiency, or undermining out-group efficiency are two means toward the same end (De Dreu et al., 2014a).

The possibility that self-sacrificial in-group cooperation and out-group aggression co-evolved (henceforth parochial altruism; Bowles, 2009) fits extant work showing that intergroup competition motivates individuals to make costly contributions to their in-group (e.g., Manson and Wrangham, 1991; Erev et al., 1993; Bornstein and Ben-Yossef, 1994; Bornstein et al., 2002; Bornstein, 2003; Bornstein and Gilula, 2003; Wildschut et al., 2003; Reeve and Holldobler, 2007; Abbink et al., 2012; Bohm and Rockenbach, 2013). It also is key in evolutionary models such as group selection and gene-culture co-evolution theory that argue that parochial altruism evolved because of impactful hostile intergroup encounters throughout human evolutionary history (Boyd and Richerson, 1982; Alexander, 1990; Choi and Bowles, 2007; Efferson et al., 2008; Bowles, 2009).

If such evolutionary perspectives on parochial altruism hold, individual propensity for parochial altruism may be sustained by evolutionary ancient neural circuitries involved in affective responding and intuitive decision making. Indeed, there is some work showing that cooperation and trust rests, in part, on sub-cortical neural circuitries recruited for affective and intuitive rather than deliberative, controlled decision making (Rilling and Sanfey, 2011). For example, parochial altruism is modulated by hypothalamic oxytocin that acts on the amygdala-hippocampal circuitries more than on prefrontal brain areas involved in impulse-control and deliberation (De Dreu et al., 2010, 2012, 2014b; Baumgartner et al., 2014; Carter, 2014; De Dreu and Kret, 2015; Ma et al., 2015). Neuro-imaging studies of individual tendencies to discriminate between in-group and out-group confirm that both categorization of self and others into in- and out-groups as well discriminatory preferences for the in-group over the out-group are fast and modulated by sub-cortical brain structures disconnected from executive control and cognitive monitoring (Hein et al., 2010; Baumgartner et al., 2014; Cikarna and Van Bavel, 2014; Kret et al., 2015). In addition to this, there is some evidence to suggest that cooperation in general, and parochial altruism in particular, may be fast and intuitive, rather than deliberated. From studies on public goods provision we know that cooperative individuals decided more quickly than those who withheld cooperation, and that individuals in which an intuitive mindset was primed cooperated more than those in which a deliberation mindset was activated (Rand et al., 2012). Moreover, demotivating and/or disabling deliberation by having participants perform a cognitively taxing Stroop task prior to decision making, amplified both spiteful rejection of other’s unfairness and benign reciprocation of trust (Halali et al., 2013).

Our first goal here was to examine the possibility that, consistent with the above works, self-sacrifice is fast and intuitive, rather than deliberated and calculative. Prior to decision making, participants performed a difficult and cognitively taxing (vs. easy) Stroop Task (Stroop, 1935). There is good evidence that performing a cognitively taxing task prior to decision making reduces the ability and/or motivation to exert cognitive control (Muraven et al., 2006; Baumeister and Vohs, 2007; Inzlicht and Schmeichel, 2012). Both possibilities suggest that following a cognitively taxing task, decision making becomes more impulsive, and intuitive (Hagger et al., 2010). Accordingly, we expected (i) more parochial altruism when decision making is intuitive, and (ii) faster decisions to be associated with more self-sacrifice.

We pursued two additional goals. First, the work on parochial altruism allows for the possibility that individuals self-sacrifice to serve the in-group (Brewer, 1999; Halevy et al., 2008), or to hurt the out-group (Brewer, 1999), or to simultaneously serve the in-group and hurt the out-group. Specifically, earlier work using experimental games, such as the intergroup prisoner’s dilemma (Bornstein, 2003), showed that intergroup competition motivates individuals to extend cooperation toward their in-group, yet such cooperation not only benefitted their in-group but also, at the same time, hurt the out-group (Halevy et al., 2008). Halevy et al. (2008) designed a Intergroup Prisoner’s Dilemma-Maximizing Differences (IPD–MD) game to examine whether such simultaneously benefitting the in-group and hurting the out-group, in fact, primarily reflects a desire to serve the in-group, and their results indeed supported such a proposition—in intergroup settings, individuals contributed more to a within-group pool that benefitted their in-group, than to a between-group pool that benefitted their in-group and simultaneously hurt their out-group. This result fits the outcomes of a recent meta-analysis (Balliet et al., 2014), along with a growing body of primary studies (Halevy et al., 2008, 2010, 2012; De Dreu, 2010; De Dreu et al., 2010; Buttelmann and Böhm, 2014) that in intergroup settings individuals are motivated more by a desire to benefit the in-group than by a desire to hurt the out-group.

Using the experimental set-up designed by Halevy et al. (2008), we explored whether cognitive taxation increases contributions to the within-group pool, or whether it increases parochial altruism (the between-group pool). Second, we examined whether parochial altruism is stronger among individuals who value fairness and cooperation (henceforth “pro-socials,” Van Lange, 1999), compared to those who value personal outcomes, and relative gain (henceforth “pro-selfs”). While evolutionary perspectives on parochial altruism are silent about the possibility that individual differences in value orientation impacts parochial altruism, several studies indicate that in intergroup competition, pro-social individuals display stronger parochial altruism, and a desire to benefit the in-group in particular, than pro-selfs (De Dreu, 2010; De Dreu et al., 2010; Abbink et al., 2012; Aaldering et al., 2013). We expected to replicate this finding, and explored whether cognitive taxation interacts with social value orientation in driving parochial altruism.

## Materials and Methods

### Participants, Ethics, and Experimental Design

Participants were recruited through the web-portal of the Psychology Research Institute of the University of Amsterdam. Based on our earlier work (De Dreu, 2010; De Dreu et al., 2010) we set a required sample size of 100, and recruited a total *N* = 111 (35 males and 76 females; *M*_age_ = 21.44, SD = 2.66) to participate in a study on human decision making. Participants received a €7 show-up fee, and the possibility to earn extra money through decision making (actual extra earnings were *M* = €5.75, range €0–€15). The study was approved by the Psychology Ethics Committee (file number 2012-WOP-2501), and participants provided written informed consent prior to the study. They were paid and debriefed upon completion of the study.

The design involved a 2 (Cognitive Taxation: Yes/No) × 2 (Social Value Orientation: Pro-social/Pro-self) between-subjects design. Participants were randomly assigned to the first factor; social value orientation was a *post hoc* blocking factor. Dependent variables were the time taken to decide (log-transformed), self-sacrifice (money contributed) as well as its decomposition in within-group and between-group contribution (the latter counting as parochial altruism). A post-experimental questionnaire assessed expectations that in-group members contributed to the within-group pool and the adequacy of the cognitive taxation manipulation.

### Procedure and Measures

**FIGURE 1:**
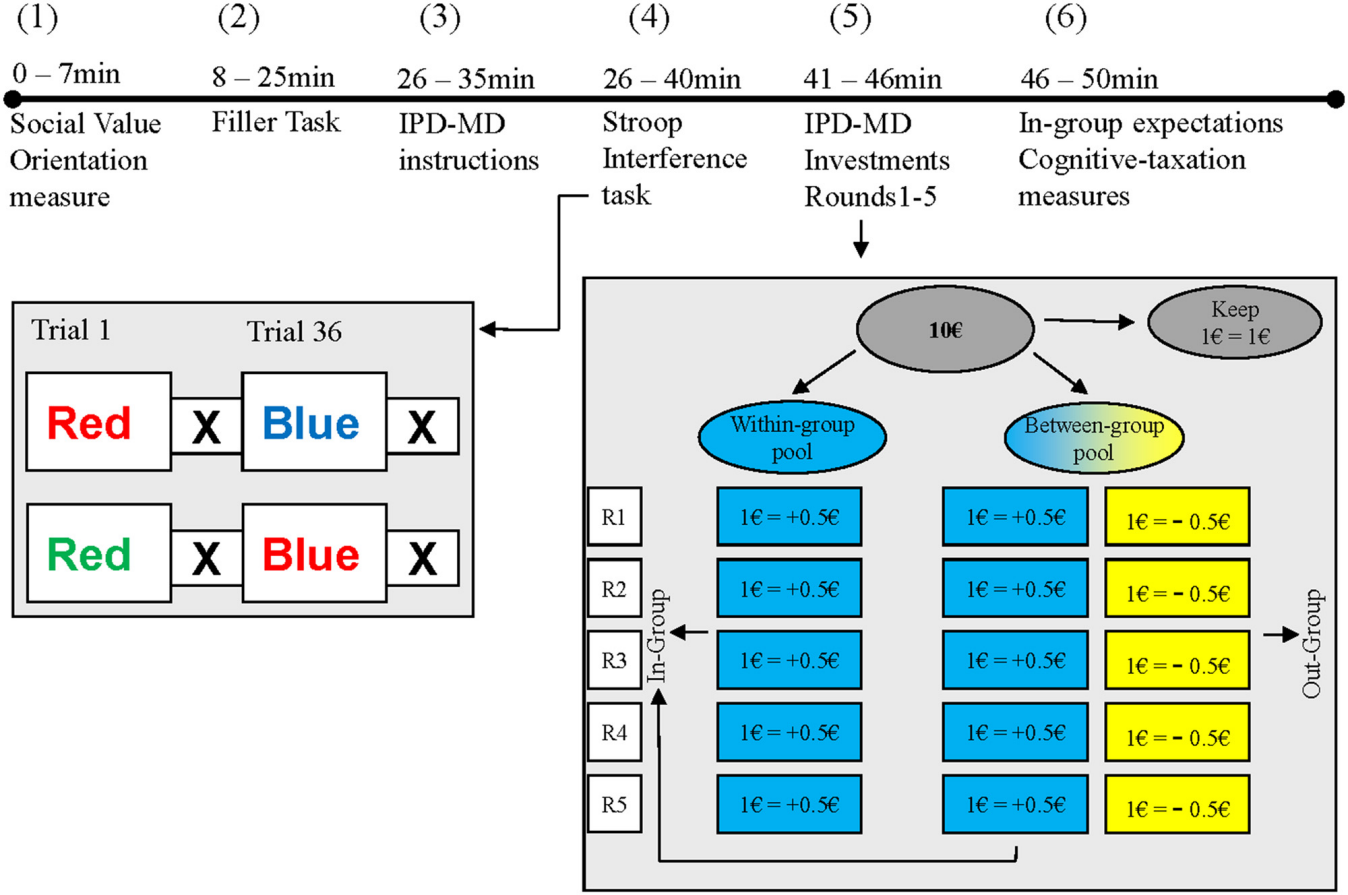
**Overview of experimental procedures and timeline**. Following assessment of (1) social value orientation using nine decomposed games, subjects (2) completed irrelevant filler-tasks, were (3) organized in two three-person groups and explained the IPD–MD, before they (4) performed either a high or low taxing Stroop Interference Task to tax cognitive resources or not, and then (5) made five IPD–MD decisions, (6) indicated their in-group expectations, and finished with cognitive-taxation measures. Coloring is for illustrative purposes only.

Experimental sessions involved groups of six individual participants. Upon arrival in the laboratory, participants were seated in individual cubicles that prevented them from seeing or hearing others. The experimenter unlocked the computer, and left. From that point onward, the experiment was computer-guided. **Figure 1** gives a schematic representation of the experimental tasks and time-lines. As can be seen, participants first completed a measure to assess their social value orientation, then performed a series of filler tests that contained no experimental manipulations, received instructions for the IPD–MD and performed a more or less cognitively taxing Stroop task to manipulate cognitive taxation. Then they made decisions in the IPD–MD, responded to a short questionnaire, and were debriefed and dismissed.

To assess social value orientation, we used a standard decomposed triple dominance measure that is widely used and well-validated (Van Lange and Kuhlman, 1994; De Dreu and Van Lange, 1995; Van Lange, 1999; Aaldering et al., 2013; also see Murphy and Ackermann, 2014). In each of nine decomposed games, subjects could choose from three different distributions of points to themselves and another person that they did not know and would not meet. An example is the decision between Option 1 [560 to You; 300 to Other], Option 2 [500 to You; 100 to Other], and Option 3 [500 to You; 500 to Other]). Option 1 reflects individualism because one’s own outcomes (560) exceed those in Option 2 (500) or Option 3 (500). Option 2 reflects competition, because it provides a greater advantage over the other’s outcomes (500 - 100 = 400) than Option 1 (560 - 300 = 260) or Option 3 (500 - 500 = 0), and Option 3 reflects a pro-social orientation because it provides a larger joint outcome (500 + 500 = 1000) than either Option 1 (560 + 300 = 860) or Option 2 (500 + 100 = 600). To be classified as pro-social (pro-self) participants had to choose consistently in at least six of the nine games. Ninety-five subjects were classified as pro-social (*N* = 45) or pro-self (*N* = 50; 45 were individualistic, and five were consistently competitive). The remaining subjects were unclassifiable and dropped from the analyses.

Following the decomposed game measure, subjects continued with a series of unrelated filler-tasks (surveys about attitudes regarding various health issues) to create a gap between the measurement of social values and the IPD–MD (Halevy et al., 2008) we used to study parochial altruism. After 25 min, the computer ended the filler-tasks and introduced the IPD–MD (Halevy et al., 2008, 2010, 2012; De Dreu, 2010). Subjects were told that they would make decisions involving the participant’s own group (denoted as “Team C2”), and another three-person group (denoted as “Team H5”; labeling was counterbalanced but never had effects and is further ignored). Participants were informed that groups were composed on the basis of the order in which they had signed up for the experiment, and that most, but not necessarily all, group members were currently present in the laboratory. They were also told that they would not know who was in their group or who was in the other group (Halevy et al., 2008; De Dreu, 2010).

Hereafter, computer instructions stated that for group decision making, each individual group member would receive an endowment of €10, which was in addition to individuals’ participation fee. Next, we explained that each Euro kept was worth €1 for the individual; each Euro that was contributed to the within-group pool added €0.50 to each in-group member including the contributor; each Euro that was contributed to the between-group pool added €0.50 to each in-group member including the contributor and, in addition, also subtracted €0.50 from each out-group member. The amount contributed to the within-group pool reflects “in-group cooperation,” the amount contributed to the between-group pool reflects parochial altruism (or “out-group hate” per Halevy et al., 2008). The instructions were in neutral language and there was no mention of the words cooperation or competition. Participants were assured that their decisions would remain completely confidential, and solved a quiz that tested their understanding of the rules of the game [i.e., we provided a series of possible group-level investments (e.g., you keep three, invest five in the within-group pool, and invest two in the between-group pool; all other members of your Team, and those of the other Team keep their endowments and make no investment: how much do you receive? How much does another member of your Team receive?)]. Analyses of their responses showed that all subjects understood the game.

Following the IPD–MD instructions, participants were given a short task intended to lower cognitive control and increase intuitive decision making. The task was modeled after the Stroop Interference Task (SIT; Stroop, 1935; Hagger et al., 2010; Halali et al., 2013, 2014). Subjects were shown a color word on their screen (e.g., “blue”) that was written in a particular color (e.g., blue) and had to indicate as fast and accurately as possible what the color of the word was. In the high taxing condition, we provided thirty-six inconsistent stimuli (i.e., color words written in a different color, e.g., “blue” written in red). Studies have shown that responding to inconsistent stimuli is cognitive taxing and reduces the ability and motivation to engage executive and impulse-control (Stroop, 1935; Hagger et al., 2010). Two subjects in this condition indicated they were color-blind and dropped from the analyses (as they were also unclassifiable regarding their social value orientation, this did not further reduce our final sample size). In the low-taxing condition, we provided 36 consistent stimuli (i.e., color words written in the same color, e.g., “blue” written in blue).

Immediately following the low or high taxing SIT, participants were asked to indicate how much of their €10 they contributed to the within-group pool, how much they contributed to the between-group pool, and how much they kept for themselves. Participants were asked to make their allocation decision five times and told that one of these decisions would be randomly drawn for payout. Participants knew that no feedback about others’ choices would be given. Investment decisions were averaged across the five rounds (α = 0.93 for within-group investments; α = 0.94 for between-group investments; *r*_within-between_ = -0.396, *p* = 0.001). In addition to recording investments, we measured time in seconds taken to decide how much participants kept to themselves (i.e., the reverse of self-sacrifice; α = 0.88). Decision time was log-transformed to meet the requirements for parametric testing (analyses using the observed data permitted the same conclusions).

Following IPD–MD investments we measured in-group expectations by asking subjects to indicate the extent to which they expected fellow in-group members to contribute to the within-group pool (1 = not at all; 7 = very much). To verify the effectiveness of the cognitive-taxation manipulation, participants were given an (unknowingly) unsolvable anagram which they were asked to solve. Time was unlimited and at subjects’ discretion, and we assessed how much time they persisted on this task. Then participants indicated how alert and concentrated they felt (both 1 = not at all, to 5 = very much; *r* = 0.421, *p* < 0.01). This completed the experiment.

## Results

### Manipulation Checks

Participants given the high taxing SIT gave up earlier on trying to solve an (unknowingly) unsolvable anagram [*M*_SIThightaxing_ = 46.94 s vs. *M*_SITlowtaxing_ = 72.60 s, *F*(1,91) = 11.19, *p* = 0.001]. They also felt less alert and concentrated [combined *M*_SIThightaxing_ = 2.47 vs. *M*_SITlowtaxing_ = 2.95, *F*(1,91) = 5.483, *p* = 0.021]. From these results, we conclude that those given the high taxing SIT had lowered cognitive control than those given the low taxing SIT.

### Decision Making

**FIGURE 2:**
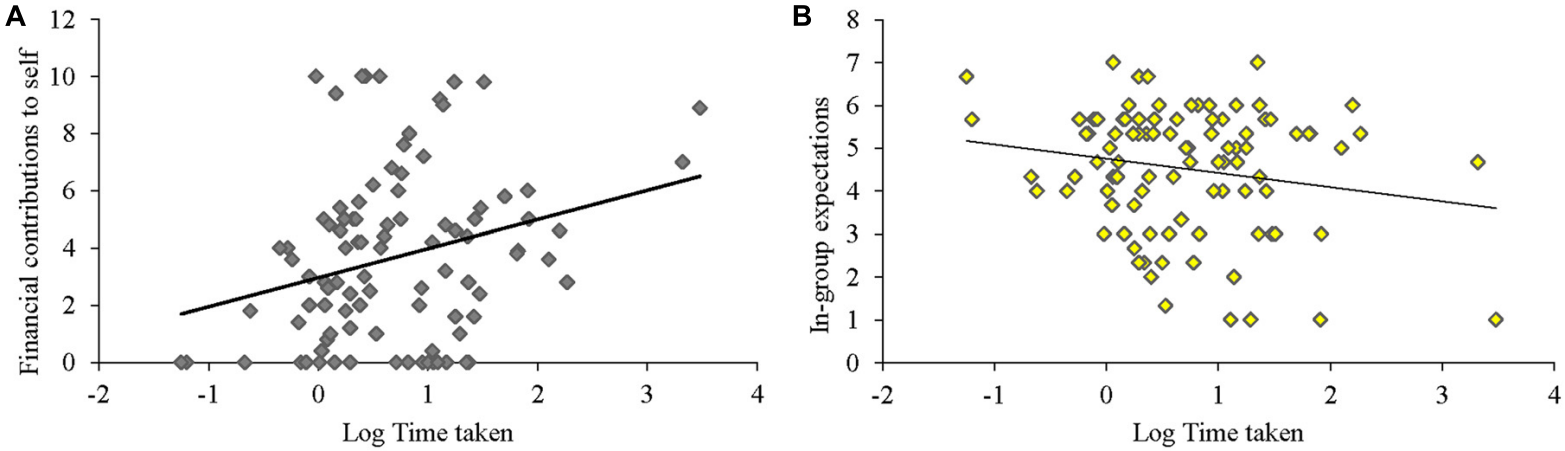
**Longer time used when deciding how much to keep to oneself (in seconds, log-transformed) associates with more selfish decision-making and less positive expectations of in-group members. (A)** Time (log-transformed seconds) taken to decide how much to keep positively relates to contribution to self. **(B)** Time (log-transformed seconds) taken to decide how much to keep negatively relates to in-group expectations.

Consistent with the possibility that self-sacrifice is intuitive, self-reported alertness and concentration was positively correlated with the amount participants kept for themselves, *r*(95) = 0.231, *p* = 0.024. Furthermore, **Figure 2A** shows that the time it took subjects to decide how much to keep to themselves positively predicted how much they kept to themselves, *r*(95) = 0.272, *p* = 0.008. Decision time was negatively related to within-group contributions, *r*(95) = -0.220, *p* = 0.033, and unrelated to parochial altruism, *r*(95) = -0.067, *p* = 0.51. Finally, **Figure 2B** shows that time to ponder how much to keep was negatively related to in-group expectations, *r*(95) = -0.185, *p* = 0.078 (marginal). These results are consistent with the idea that selfish behavior is deliberated whereas self-sacrifice and assessments of in-group members’ cooperation are relatively fast and intuitive.

**FIGURE 3:**
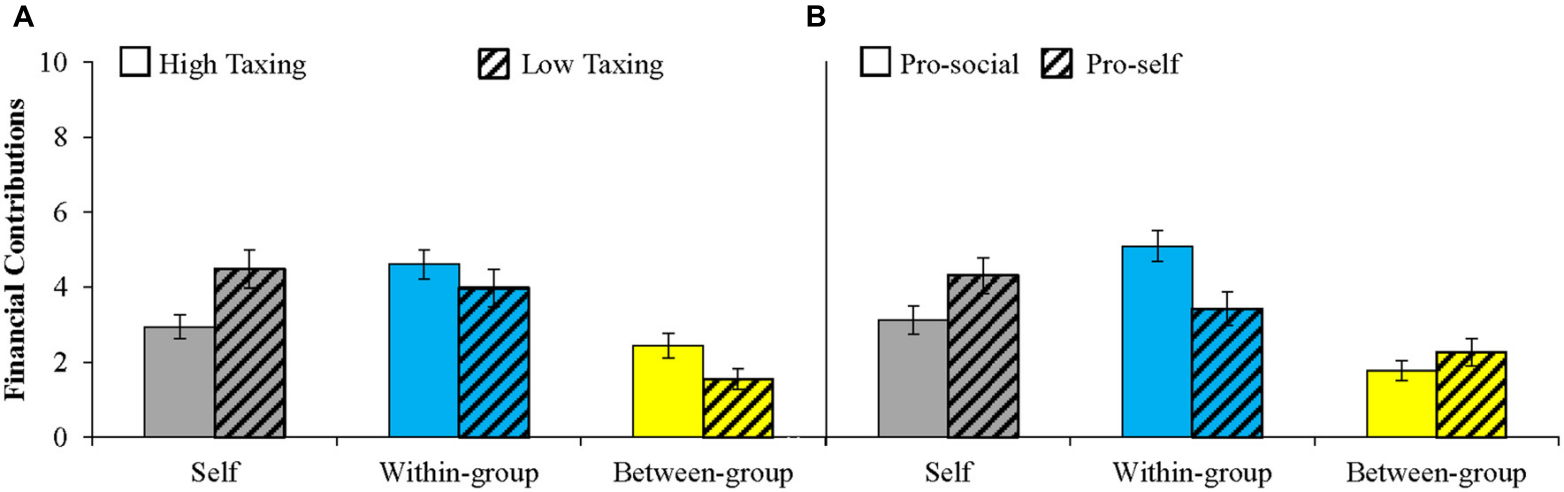
**Averaged Intergroup Prisoner’s Dilemma–Maximizing Differences Game (IPD–MD) contributions to self, within-group pool, and between-group pool (parochial altruism; range 0–10; means displayed ±1 SE). (A)** Compared to individuals in the low taxing condition (*N* = 46), individuals in the high taxing condition (*N* = 49) self-sacrifice, and display parochial altruism. **(B)** Compared to pro-selves (*N* = 50), pro-social individuals (*N* = 45) self-sacrifice, contribute more to the within-group pool, and display similar levels of parochial altruism.

Intergroup Prisoner’s Dilemma–Maximizing Differences Game investments were averaged and analyzed in a 2 (Yes/No cognitive taxing SIT) × 2 (pro-social/pro-self) × 3 (pool: self/within-group/between-group) mixed-model ANOVA with pool within-subjects. A strong main effect for Pool showed, first of all, that self-sacrifice manifested in within-group contributions more than in between-group contributions, *M* = 4.304 vs. *M* = 2.11, *F*(1,91) = 25.77, *p* = 0.0001. This effect was qualified by an interaction between cognitive taxation and pool, *F*(2,90) = 4.59, *p* = 0.013. **Figure 3A** shows that parochial altruism was stronger among high taxed (vs. low taxed) individuals, *F*(1,91) = 7.08, *p* = 0.009. Interestingly, compared to low taxed individuals, those in the high taxing condition allocated about equally to the within-group pool, *F*(1,91) = 1.01, *p* = 0.32, and more to the between-group pool, *F*(1,91) = 4.81, *p* = 0.031. Together these results suggest that lowered cognitive control following taxation increases contributions to the between-group pool, and this fits the idea that parochial altruism is more intuitive than deliberated.

Cognitive taxation did not interact with social value orientation in influencing contributions, *F*s < 1.9. However, consistent with earlier work, we observed a pool × social value orientation interaction, *F*(2,90) = 3.573, *p* = 0.032: pro-socials kept less to themselves, *F*(1,91) = 4.20, *p* = 0.043, contributed more to the within-group pool, *F*(1,91) = 7.29, *p* = 0.008, and equally to the between-group pool as pro-selves, *F*(1,91) = 1.19, *p* = 0.278 (**Figure 3B**). Both pro-selves and pro-socials contributed more than nothing to the between-group pool, *t*(44) = 6.315, *p* = 0.001, and *t*(49) = 6.833, *p* = 0.001. Together these results show that whereas pro-selves are non-cooperative toward their in-group, pro-socials benefit their in-group. Both pro-socials and pro-selves maintain a certain level of parochial altruism regardless of whether or not they are cognitively taxed.

The above analyses leave open the possibility that some participants invested exclusively in the within-group pool (and could thus be described as in-group bounded cooperators as well as universal cooperators), whereas others invested exclusively in the between-group pool (and thus are strict parochial altruists). To investigate this possibility, we classified subjects into distinct types: those who kept their entire endowment (selfish types), those who contributed exclusively to the within-group pool (in-group cooperators), those who contributed to both the within- and between-group pool (ambivalent parochial altruists), and those who contributed exclusively to the between-group pool (strict parochial altruists). **Table 1** shows the breakdown of types by cognitive taxation, with the 4 × 2 interaction being, χ^2^(3, *N* = 95) = 7.115, *p* = 0.068. As can be seen, very few participants never (across five investment decisions) contributed anything (selfish types) and very few contributed exclusively to the between-group pool (strict parochial altruists). Across the five investment decisions, most participants did invest (>0) in either the within-group pool (in-group cooperators) or in both the within-group and the between-group pool (ambivalent parochial altruists). As can be seen also, especially ambivalent parochial altruists emerge under cognitive taxation. This resonates with the above analysis showing that cognitive taxation boosts parochial altruism. It suggests that such a boost is not a reflection of a shift toward strict parochial altruism, but instead a muddying of cooperation with the in-group and aggression toward the rival out-group.

**Table 1:** Contributor types broken down by cognitive taxation.

		Contributor type		
Cognitive taxation	Selfish	In-group cooperator	Ambivalent parochial altruists	Strict parochial altruists
Yes (*N* = 49) No (*N* = 46)	1 (2%) 3 (7%)	12 (24%) 16 (35%)	35 (71%) 22 (48%)	1 (2%) 5 (10%)

*Entries are observed number of subjects (row-based percentages in brackets). Selfish types keep their endowment; In-group cooperators contributed (>0) exclusively to the within-group pool; ambivalent aarochial altruists contributed (>0) to both the within- and between-group pool; strict parochial altruists exclusively contributed (>0) to the between-group pool*.

We also examined the break-down of types by social value orientation, which was not significant, χ^2^(3, *N* = 95) = 4.848, *p* = 0.183. Among pro-social (pro-self) individuals, 27% (32%) were in-group cooperators, and 56% (64%) were ambivalent parochial altruists. This suggests that the above findings for social value orientation reflect the strength of motivation—the extent to which people decide to contribute—rather than its strict directionality.

### In-Group Expectations

Expectations of in-group members contributions to the within-group pool was positively related to self-sacrifice, *r*(95) = 0.445, *p* = 0.001, especially to contributions to the within-group pool, *r*(95) = 0.469, *p* = 0.001, and not to contributions to the between-group pool, *r*(95) = -0.043, *p* = 0.677). A 2(Yes/No cognitively taxing SIT) × 2(pro-social/pro-self) between-subjects ANOVA on in-group expectations showed less positive expectations among individuals in the high cognitive taxing condition (*M* = 4.809) compared to those in the low cognitive taxing condition (*M* = 4.267), *F*(1,91) = 4.32, *p* = 0.040. Pro-social individuals also displayed (somewhat) more positive expectations (*M* = 4.753) than pro-selves (*M* = 4.211), *F*(1,91) = 2.86, *p* = 0.094. The cognitive taxation × social value interaction was not significant, *F*(1,91) = 0.73, *p* = 0.395. In short, cognitively taxed individuals, and to a lesser extent those with a pro-social value orientation, had more positive expectations of their in-group members.

## Discussion and Conclusion

Human cooperation and self-sacrifice may emerge partly because it provides survival benefits in intergroup competition and, therefore, often is in-group bounded and parochial (Bornstein, 2003; De Dreu et al., 2014a). Heretofore unanswered was whether such self-sacrifice in intergroup competition was a deliberated, calculated response to pressing conflicts between personal and group interests or, instead, a relatively automatic and intuitive decision. Present findings align with the latter possibility—whereas selfishness appeared to be the slower and thus more deliberated response, self-sacrifice appeared to be fast and thus more intuitive. Indeed, self-sacrificial contributions were released when individual’s cognitive control was taxed. This resonates with recent work in neurobiology and cognitive psychology (Kosfeld et al., 2005; De Dreu et al., 2010, 2012; Rand et al., 2012; Van Honk et al., 2012) and supports the conjecture that cooperation is oftentimes intuitive, in-group-bounded, and potentially sharp-edged.

Current findings inform neo-classical rational choice theories that view humans to be motivated to maximize personal, short-term gain. Our results, and those of others (Rand et al., 2012; Halali et al., 2013, 2014), support the idea that human cooperation is intuitive rather than calculated. Furthermore, the conjecture that cooperation is primarily geared at benefiting one’s in-group while simultaneously hurting the out-group fits evidence that brain circuitries involved in empathy and other-concern operate much stronger when targets are classified as in-group rather than out-group (Harris and Fiske, 2006; Hein et al., 2010; Cikara et al., 2011; Baumgartner et al., 2012).

We studied intuitive cooperation in the presence of an out-group that was enabled to hurt the in-group. Collective action oftentimes takes place in absence of rivaling out-groups, in which case in-group boundedness and universalism as foci of self-sacrifice cannot be separated (Wit and Kerr, 2002; De Dreu and Balliet, 2015). Perhaps, the default, intuitive response in social dilemmas is to cooperate, with such cooperation becoming parochial when out-group competition is present. Perhaps, however, the default, intuitive response is to serve one’s in-group, which translates into universalism when no other option to serve one’s in-group is available (Burton-Chellew and West, 2012; also see De Dreu and Balliet, 2015). We call for new research mapping situations ranging from inter-group competition being fully absent to being strongly present, to further understand when and why human cooperation evolved into an automated, intuitive response that is released rather than blocked by impeded impulse-control.

The impulse to benefit one’s in-group was triggered here by a simple task known to undermine executive functioning (Stroop, 1935). We note that because of time constraints, multiple tasks awaiting attention, ambient noise, and social pressures, the human capacity to deliberate and to think deeply before acting is constantly challenged (Kruglanski et al., 2006; De Dreu et al., 2008). However, it is important to note that questions have been raised about depletion as a manipulation of intuitive vs. deliberativeness. For example, looking at altruism in the Dictator Game, depletion typically reduces altruism (Balliet and Joireman, 2010; Xu et al., 2012; Halali et al., 2013), whereas cognitive load (which is a direct manipulation of cognitive resources) typically increases altruism (Roch et al., 2000; Cornelissen et al., 2011; Schulz et al., 2014). A recent study by Rand et al. (2014) relates to the current findings, in that they also examined how decision making constraints—time pressure during decision making in their case, compared to cognitive taxation prior decision making in the current study—influenced cooperation in intergroup settings. Experiment 1 involved a two-person prisoner’s dilemmas between a participant and an antagonist said to either prefer the same presidential candidate (e.g., Obama), or the opposing candidate (e.g., Romney). Results showed that time pressure facilitated cooperation, and that people cooperated more with someone that preferred the same presidential candidate, rather than the opposing candidate; no interaction among time pressure and antagonist’s political preference was observed. Our finding that cognitive taxation increases contributions not only fits this, but also clarifies that increased cooperation with someone sharing the same political preference is motivated by a desire to benefit the in-group more than a desire to hurt the out-group (also see Balliet et al., 2014). This convergence notwithstanding, new research using a different manipulation is required to conclusively settle whether time constraints and cognitive taxation impact cooperation because of their shared impact on intuitive versus more deliberated decision making. Such new research should include sophisticated measures also of depletion and impulse-control, akin to the measures used here that showed that the cognitive taxing manipulation not only affected self-sacrifice and parochial altruism, but also decision time, post-decision making task-persistence, and self-reported fatigue and alertness.

Current findings suggest that cognitively challenging contexts lower self-control and facilitate rather than impede cooperation and collective action. Such cognitively challenging settings can also trigger negative behaviors toward rival out-groups. Displays of parochial altruism not only increase in-group status relative to the out-group but also negative emotions and aggressive responses among maltreated out-group members (Dovidio and Gaertner, 2010; De Dreu et al., 2014b). Ironically, then, whereas humans may have the evolved intuition to cooperate, it is unlikely such intuition brings world peace closer.

## Author Contributions

BD, FTV, and CKWDD conceived of the study; BD and FTV conducted the study; BD and CKWDD analyzed the data; and CKWDD wrote the paper.

## Conflict of Interest Statement

The authors declare that the research was conducted in the absence of any commercial or financial relationships that could be construed as a potential conflict of interest.
